# Imaging findings in a patient with suspected vaccine induced immune thrombotic thrombocytopenia

**DOI:** 10.1259/bjrcr.20210138

**Published:** 2021-10-06

**Authors:** Nikhil Kadam, Kumar VM Ramavathu, Namita Kamath, Kaung Kyaw Min

**Affiliations:** 1 Internal Medicine Trainee, Southend University Hospital, Mid and South Essex NHS Trust, Southend-on-Sea, United Kingdom; 2 Southend University Hospital, Mid and South Essex NHS Trust, Southend-on-Sea, United Kingdom

## Abstract

Covid-19 vaccine was developed in response to the SARS Cov2 pandemic. Despite the effectiveness of the vaccine, various complications have been reported after vaccination.

We present the case of a 55-year-old patient with post-vaccination complication. The patient was vaccinated with ChAdOx1 nCov-19 Vaccine and 2 weeks later presented with headache, confusion and abdominal pain for 1 week duration. Clinical examination demonstrated reduced Glasgow Coma Scale (GCS), reduced muscle power bilaterally and dysphasia. Blood test showed thrombocytopenia, high titres of D-Dimer and mildly raised INR.

The CT scan of the head showed a fairly large left temporoparietal intracranial hemorrhage with midline shift and subsequent CT venogram demonstratedthrombosis of the left transverse and sigmoid dural venous sinuses. CT scan of the abdomen and pelvis showed thrombosis of the portal and hepatic veins and multiple infarcts of the liver, left kidney and lingular segment of the partially imaged lungs (Figure 2).

Patient tested positive for antibodies directed against platelet factor-4 and was treated for vaccine induced thrombotic thrombocytopenia. Treatment included Intravenous immunoglobulin, Fresh Frozen Plasma, non-heparin based anticoagulant and required care in tertiary center.

Incidence of vaccine-induced immune thrombotic thrombocytopenia is unknown and strongly mimics autoimmune heparin-induced thrombocytopenia with typical clinical features of thrombocytopenia and thrombosis. Of the reported cases, the common imaging finding is thrombosis in various sites such as cerebral venous thrombosis, portal vein thrombosis, pulmonary embolism and ischemic stroke.

## Introduction

Covid-19 vaccine was developed in response to the SARS Cov2 pandemic. Despite the effectiveness of the vaccine, various complications have been reported after vaccination which may vary with type of vaccine. We present the case of a 55-year-old patient with post-vaccination complication.

## Case report and imaging findings

The patient was vaccinated with ChAdOx1 nCov-19 Vaccine and 2 weeks later presented with headache, confusion and abdominal pain for 1 week duration. Clinical examination demonstrated reduced Glasgow Coma Scale (GCS), reduced muscle power bilaterally and dysphasia. Blood test showed thrombocytopenia (73 × 10^9^ l^−1^), high titres of D-Dimer (6177 ng ml^−1^), elevated prothrombin time (20.2 s) and alanine aminotransferase (537 U l^−1^).

**Figure 1.**
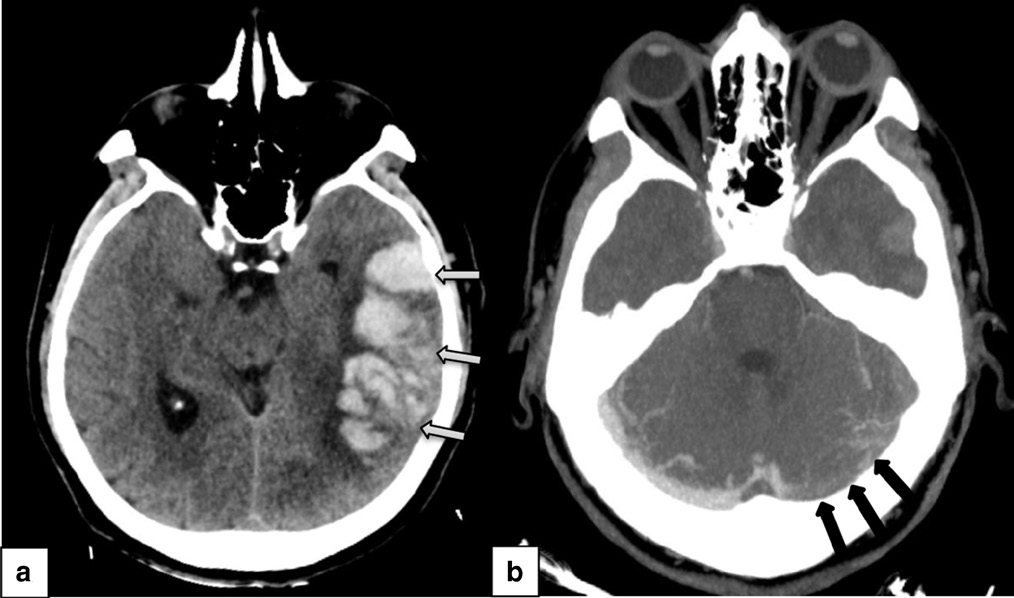
CT scan of the head (a) shows a large left frontotemporoparietal hemorrhage (grey arrows) with surrounding edema causing mass effect over the left lateral ventricle and surrounding sulci. CT venogram (B) shows thrombosis of the left transverse sinus (black arrows).

**Figure 2.**
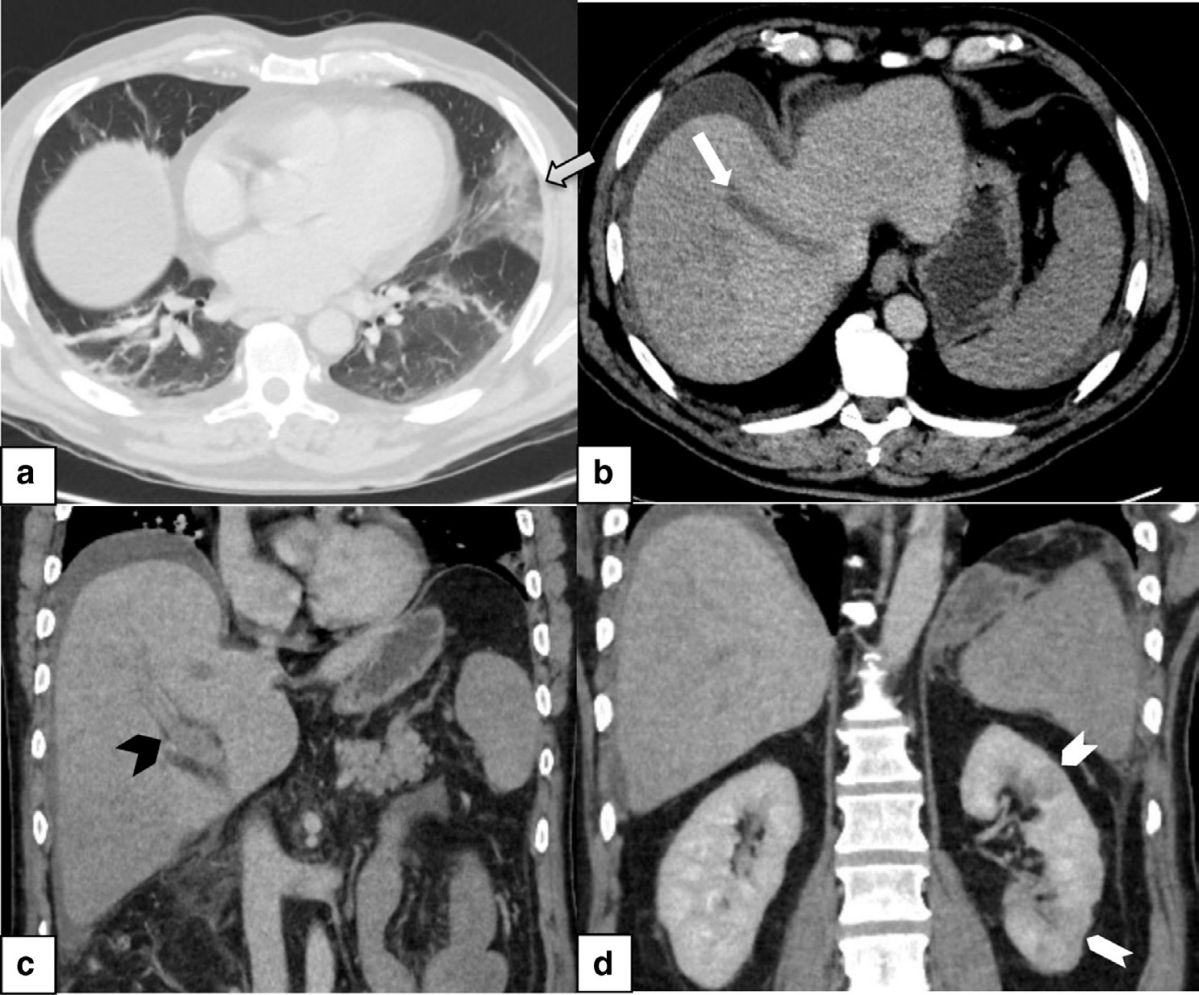
CT in portovenous phase shows (a) a wedge-shaped infarct (grey arrow) in the linguar segment of the lung (b) axial section of the liver with a thrombus in the middle hepatic vein (white arrow). Coronal images of the abdomen demonstrate (c) portal vein thrombosis (black arrowhead) and (d) infarcts in the left kidney (white arrowheads).

The CT scan of the brain showed a left temporoparietal intracranial hemorrhage measuring 7 × 4.4 x 5 cm with midline shift and subsequent CT venogram demonstrated filling defects in the left transverse and sigmoid venous sinuses consistent with thrombosis. (Figure 1). CT scan of the abdomen and pelvis showed thrombosis of the portal and hepatic veins and multiple infarcts of the liver, left kidney and lingular segment of the partially imaged lungs (Figure 2).

The patient tested positive for antibodies directed against platelet factor-4 (0.5 units) and was diagnosed with vaccine-induced thrombotic thrombocytopenia. The treatment included Intravenous immunoglobulin, Fresh Frozen Plasma, non-heparin based anticoagulant and plasma exchange at a tertiary care center.

## Discussion

Post-vaccination entity termed as vaccine-induced immune thrombotic thrombocytopenia (VITT) appears to be a phenomenon associated with rare case of serious blood clots, including blood clots in the brain and other critical organs. Incidence of VITT is unknown but case reports and case series of cerebral venous thrombosis (CVT) occurring within 4–42 days of ChAdOx1 nCoV-19 vaccination, a vaccine produced by AstraZeneca (AZ), have been published.^1–6^ Similar cases were reported in association with the Ad26.COV2.s Johnson & Johnson/Janssen (JJ) vaccine.^7–9^

The most common clinical presentations of patients with VITT were noted to be focal deficit, altered mental state, reduced consciousness levels, headache and occasionally abdominal symptoms like non-specific abdominal pain. CVT was confirmed in these cases by CT with CT venography or MR with MR venography as part of their initial investigations.^10,11^

CT brain scan findings in reported cases include CVT and cerebral hemorrhage. In those presenting with abdominal symptoms, the key imaging findings were portal-vein thrombosis extending to splenic and upper mesenteric veins along with diffuse gastrointestinal bleeding with reduced perfusion of the intestinal wall.^1^

Additional tests required in the diagnosis of VITT include complete blood counts, blood film, coagulation studies like D-Dimers, fibrinogen, PT and aPTT. If there is thrombocytopenia (<150×10^9^/L) or abnormal results of coagulation tests, a heparin-induced thrombocytopenia (HIT) PF4 Antibody ELISA test should be performed and, if possible, confirmed by functional tests^12^^12–14^^[Bibr b14]^ .

Most accepted current diagnostic criteria for VITT includes - COVID vaccine 4–42 days prior to symptom onset, venous or arterial thrombosis (commonly cerebral or abdominal) confirmed on imaging, thrombocytopenia, markedly elevated D-Dimer and a positive HIT ELISA test.^15^ This entity mimics autoimmune heparin-induced thrombocytopenia with typical clinical features of thrombocytopenia and thrombosis.^1,6^

The published and reported cases so far have imaging findings of thrombosis in varied sites such as cerebral venous thrombosis, portal vein thrombosis, pulmonary embolism, Lower limb deep vein thrombosis and ischemic stroke along with other positive biochemical findings of high D-Dimers, low platelet counts and Positive PF4 “HIT”ELISA antibodies.^1–9,16^

## Learning points

- VITT is a new syndrome associated with adenoviral-vectored Covid vaccine.
- Most common presentations include symptoms of thrombosis and thrombocytopenia.
- Investigations involve full blood count, coagulation study, PF-4 antibodies and imaging to confirm the thrombosis and to look for the extent of the involvement.
- Imaging undertaken in suspected cases should be based on the clinical presentation of the patient – commonly are CT brain & venogram (CNS Symptoms) and CT abdomen (abdominal symptoms).
